# Redox Network Failure in Chronic Kidney Disease: Hydrogen Sulfide Deficiency, Reactive Sulfur Species Dysregulation and the Uremic Toxin–AhR–Mitochondrial Axis

**DOI:** 10.3390/antiox15060746

**Published:** 2026-06-12

**Authors:** Kuo-Cheng Lu, Chia-Chao Wu, Te-Chao Fang, Yi-Chou Hou, Cai-Mei Zheng, Chien-Lin Lu

**Affiliations:** 1 Division of Nephrology, Department of Medicine, Taipei Tzu Chi Hospital, Buddhist Tzu Chi Medical Foundation, New Taipei City 23142, Taiwan; tch33730@tzuchi.com.tw; 2 Division of Nephrology, Department of Internal Medicine, Fu Jen Catholic University Hospital, Fu Jen Catholic University, New Taipei City 24352, Taiwan; 3 Division of Nephrology, Department of Internal Medicine, Tri-Service General Hospital, National Defense Medical Center, Taipei 11490, Taiwan; 4 Division of Nephrology, Department of Internal Medicine, School of Medicine, College of Medicine, Taipei Medical University, Taipei 11031, Taiwan; 5 Division of Nephrology, Department of Internal Medicine, Taipei Medical University Hospital, Taipei Medical University, Taipei 11031, Taiwan; 6 TMU Research Center of Urology and Kidney, Taipei Medical University, Taipei 11031, Taiwan; 7 Division of Nephrology, Department of Internal Medicine, Cardinal-Tien Hospital, New Taipei City 23155, Taiwan; 8 School of Medicine, College of Medicine, Fu Jen Catholic University, New Taipei City 24205, Taiwan; 9 Division of Nephrology, Department of Internal Medicine, Shuang Ho Hospital, Taipei Medical University, New Taipei City 23561, Taiwan

**Keywords:** aryl hydrocarbon receptor, chronic kidney disease, hydrogen sulfide, indoxyl sulfate, redox signaling

## Abstract

Chronic kidney disease (CKD) affects approximately 700 million people worldwide and is a major contributor to end-stage renal disease (ESRD), cardiovascular morbidity, and premature mortality. Although oxidative stress has long been considered central to CKD progression, conventional antioxidant strategies have not consistently improved clinical outcomes, suggesting that excess reactive oxygen species (ROS) alone cannot fully account for the underlying disease pathophysiology. Emerging evidence supports a broader paradigm of redox network failure, characterized by the disruption of coordinated signaling among ROS, nitric oxide (NO), and reactive sulfur species (RSS). Within this framework, hydrogen sulfide (H_2_S), a major endogenous RSS, functions as a key regulator of renal redox homeostasis. CKD is consistently associated with systemic and renal H_2_S deficiency, accompanied by downregulation of cystathionine β-synthase (CBS), cystathionine γ-lyase (CSE), and 3-mercaptopyruvate sulfurtransferase (3-MST), as well as impaired transsulfuration and disrupted mitochondrial sulfide oxidation. Importantly, this deficiency cannot be explained solely by reduced renal function but instead reflects active suppression of H_2_S biosynthesis. Uremic toxins, particularly indoxyl sulfate (IS), contribute to this process through activation of the aryl hydrocarbon receptor (AhR), which inhibits specificity protein 1 (Sp1)-dependent transcription of H_2_S-producing enzymes. This IS–AhR–Sp1 axis provides a mechanistic link between toxin accumulation and disruption of the sulfur arm of the redox network, amplifying oxidative stress, endothelial dysfunction, mitochondrial impairment, ferroptotic vulnerability, and fibrotic remodeling. Beyond H_2_S itself, downstream RSS, including persulfides, polysulfides, and thiosulfate, may represent the principal bioactive mediators of sulfur-dependent redox signaling, and their coordinated depletion in CKD may impair redox buffering capacity beyond what H_2_S measurement alone reflects. This review integrates current evidence to propose a conceptual model in which CKD progression involves failure of coordinated redox signaling—characterized by feed-forward network collapse and threshold-dependent transition to a self-sustaining high-ROS state—with H_2_S deficiency representing one mechanistically supported component of this broader network disruption. This framework highlights the therapeutic potential of targeting redox network restoration rather than isolated oxidative pathways in CKD.

## 1. Redox Network Failure in CKD

Chronic kidney disease (CKD) is a major global health burden, affecting approximately 700 million individuals worldwide and contributing substantially to end-stage renal disease (ESRD), cardiovascular morbidity, and premature mortality [1]. Despite diverse etiologies, a shared feature across CKD subtypes is progressive disruption of redox homeostasis [2]. Oxidative stress, defined as an imbalance between pro-oxidant generation and antioxidant defenses, has long been implicated in renal injury, vascular dysfunction, and systemic complications [3].

This framework, centered on excessive reactive oxygen species (ROS), does not fully explain persistent disease progression or the limited efficacy of antioxidant therapies. Clinical and experimental evidence suggests that nonspecific antioxidant strategies have not consistently improved renal or cardiovascular outcomes, indicating that ROS excess alone does not capture the underlying pathophysiology [4]. Redox regulation is now understood as an integrated network involving coordinated interactions among ROS, nitric oxide (NO), and reactive sulfur species (RSS) [5]. This shift emphasizes dysregulation of redox signaling rather than simple accumulation of oxidants.

Within this network, hydrogen sulfide (H_2_S), a major endogenous RSS, functions as a key regulator of renal redox homeostasis. It is produced in the kidney by cystathionine β-synthase (CBS), cystathionine γ-lyase (CSE), and 3-mercaptopyruvate sulfurtransferase (3-MST), and participates in the regulation of mitochondrial metabolism, tubular transport, and redox signaling [6]. H_2_S modulates antioxidant defense primarily through activation of nuclear factor erythroid 2-related factor 2 (Nrf2) and regulates inflammatory and fibrotic pathways [7]. Accumulating evidence indicates that CKD is characterized by systemic and renal H_2_S deficiency, associated with reduced expression of H_2_S-producing enzymes and impaired biosynthesis [8]. Importantly, this deficiency is not solely explained by reduced renal excretory function but reflects active suppression of H_2_S production. Uremic toxins, particularly indoxyl sulfate (IS), contribute to this process through activation of the aryl hydrocarbon receptor (AhR), which inhibits specificity protein 1 (Sp1)-dependent transcription of H_2_S-producing enzymes, thereby linking toxin accumulation to the disruption of sulfur-mediated redox signaling [9]. It should be noted that circulating H_2_S levels represent only one component of a broader sulfur signaling network that includes free sulfide, bound sulfane sulfur, persulfides, and oxidized sulfur metabolites such as thiosulfate; disruption across these interconnected pools may collectively contribute to impaired redox regulation in CKD [10,11].

This review proposes that H_2_S deficiency may represent functional failure of the sulfur arm of the redox network, contributing to loss of coordinated redox regulation in CKD—a framework that provides a mechanistic basis for persistent disease progression, cardiovascular vulnerability, and the limited efficacy of conventional antioxidant approaches. To develop this framework, the following sections focus primarily on molecular and experimental evidence linking H_2_S deficiency to sulfur-mediated redox dysregulation in CKD, including the IS–AhR–Sp1 axis, mitochondrial sulfide oxidation, protein persulfidation, endothelial dysfunction, and inflammatory signaling. Translational and clinical findings are incorporated to support the biological relevance of these mechanisms, while therapeutic implications are discussed from a mechanistic and translational perspective rather than as a comprehensive clinical treatment review.

This narrative review was conducted through literature searches in PubMed and Google Scholar up to March 2026 using combinations of keywords including “hydrogen sulfide,” “chronic kidney disease,” “indoxyl sulfate,” “aryl hydrocarbon receptor,” “oxidative stress,” and “redox signaling.” Priority was given to original experimental and translational studies related to renal H_2_S biology and CKD-associated redox dysregulation. Studies were included if they reported original experimental or clinical data relevant to H_2_S biology, uremic toxin signaling, or redox regulation in CKD. Review articles were included only for broad conceptual background and were not used as primary mechanistic sources.

## 2. Renal H_2_S Biology and Redox Regulation

### 2.1. Enzymatic Sources and Compartmental Control of Renal H_2_S Production

H_2_S is an endogenous gasotransmitter in the kidney, alongside NO and carbon monoxide (CO), and contributes to renal hemodynamics, tubular transport, mitochondrial metabolism, and redox regulation [6]. It is generated from cysteine and homocysteine through three enzymatic systems: CBS, CSE, and 3-MST [10]. CBS and CSE operate within the transsulfuration pathway, whereas 3-MST produces H_2_S via cytosolic and mitochondrial routes coupled to cysteine aminotransferase or D-amino acid oxidase [12,13,14,15].

The kidney is a major site of H_2_S production with distinct intrarenal distribution. CBS and CSE are predominantly expressed in proximal tubular cells, while CSE is also detected in glomerular structures, supporting compartment-specific H_2_S generation [12,16,17,18]. 3-MST contributes primarily to mitochondrial H_2_S production, particularly through the 3-MST/D-amino acid oxidase pathway [6]. H_2_S bioavailability is determined not only by enzyme expression but also by substrate availability, subcellular localization, and mitochondrial catabolism via sulfide:quinone oxidoreductase (SQR) and downstream oxidation pathways [19].

This multilayer regulation renders H_2_S production highly sensitive to metabolic stress and uremic toxins, providing a mechanistic basis for the reduction in H_2_S biosynthesis observed in CKD.

Beyond proximal tubular cells, H_2_S-producing enzymes are expressed across multiple renal cell types with distinct distributions and functional implications. CSE is the predominant H_2_S source in glomerular compartments, expressed in podocytes, mesangial cells, and glomerular endothelial cells, whereas CBS is enriched in proximal tubular epithelium and 3-MST contributes primarily through mitochondrial routes in both tubular and glomerular fractions [20,21]. This cell-type specificity confers differential vulnerability to H_2_S depletion: in mesangial cells, high-glucose conditions suppress CSE and increase transforming growth factor-beta 1 (TGF-β1)-driven matrix accumulation and proliferation, effects reversed by H_2_S supplementation [20,22]; in podocytes, CSE downregulation amplifies homocysteine- and adriamycin-induced oxidative injury and glomerulosclerosis [23]. In renal fibroblasts, H_2_S donors inhibit TGF-β1/Smad-dependent myofibroblast differentiation and collagen deposition, positioning H_2_S deficiency as a permissive factor for interstitial fibrosis independently of tubular injury [22,23]. These findings indicate that H_2_S depletion in CKD does not affect all renal cell types uniformly: the relative contribution of impaired glomerular CSE, tubular CBS suppression via the IS–AhR–Sp1 axis, and loss of fibroblast H_2_S signaling to overall disease progression likely varies by CKD etiology and stage, and cell-type-specific targeting may be required for optimal therapeutic efficacy.

### 2.2. H_2_S-Dependent Regulation of Renal Microvascular and Tubular Function

H_2_S regulates cellular signaling primarily through protein persulfidation, a reversible modification of cysteine residues that alters protein activity and interaction networks [24]. This mechanism enables H_2_S to modulate pathways governing vascular tone, ion transport, and cellular stress responses.

In the kidney, H_2_S promotes vasodilation through activation of potassium channels, thereby influencing renal blood flow and glomerular filtration [25]. It also interacts with the renin–angiotensin–aldosterone system (RAAS) and NO signaling, forming part of an integrated gasotransmitter network that regulates endothelial and microvascular function [26]. At the tubular level, H_2_S inhibits sodium transport and reduces Na^+^ reabsorption, contributing to natriuresis and fluid balance [27]. Collectively, these effects position H_2_S as an integrative regulator linking redox signaling to renal vascular and tubular function.

### 2.3. Mitochondrial Sulfide Oxidation and Oxygen-Sensitive H_2_S Signaling

H_2_S is tightly coupled to mitochondrial function in renal tubular cells, which have high energy demands. At physiological concentrations, H_2_S is oxidized via SQR-dependent pathways and donates electrons to the electron transport chain, thereby supporting adenosine triphosphate (ATP) production, particularly under conditions of limited oxygen availability [6,19]. This coupling links H_2_S metabolism directly to cellular bioenergetics and redox balance.

The renal medulla represents a physiologically hypoxic environment in which H_2_S signaling is particularly relevant. Oxygen-dependent H_2_S oxidation permits sulfide accumulation in low-oxygen regions, where it regulates medullary blood flow and tubular transport [12]. In addition, H_2_S limits mitochondrial electron leakage, reduces oxidative stress, and supports adaptive responses such as autophagy and the unfolded protein response [28]. These coordinated effects integrate perfusion, metabolism, and redox control, highlighting the vulnerability of H_2_S-dependent mitochondrial regulation to disruption in CKD. Beyond this physiological coupling, mitochondrial H_2_S catabolism proceeds through a sequentially organized oxidation system downstream of SQR. The persulfide intermediate generated by SQR—primarily glutathione persulfide (GSSH)—is subsequently oxidized to sulfite by ethylmalonic encephalopathy protein 1 (ETHE1)**,** a persulfide dioxygenase, in an oxygen-dependent reaction, and sulfite is then converted to thiosulfate by rhodanese/thiosulfate sulfurtransferase (TST) or to sulfate by sulfite oxidase [29,30]. The importance of this pathway is underscored by ETHE1 deficiency, which causes sulfide accumulation, cytochrome c oxidase inhibition, and fatal systemic disease, demonstrating that impaired sulfide oxidation is as pathologically significant as impaired biosynthesis [30]. In the context of CKD, this oxidation system may be independently compromised: in diabetic kidney disease, reduced TST expression impairs mitochondrial fatty acid oxidation and worsens tubular injury, whereas TST overexpression is protective [31]. These findings indicate that disruption of the SQR–ETHE1–TST axis in CKD may contribute to altered sulfur homeostasis through impaired clearance, complementing the biosynthetic deficits mediated by CBS, CSE, and 3-MST downregulation.

**Figure 1:**
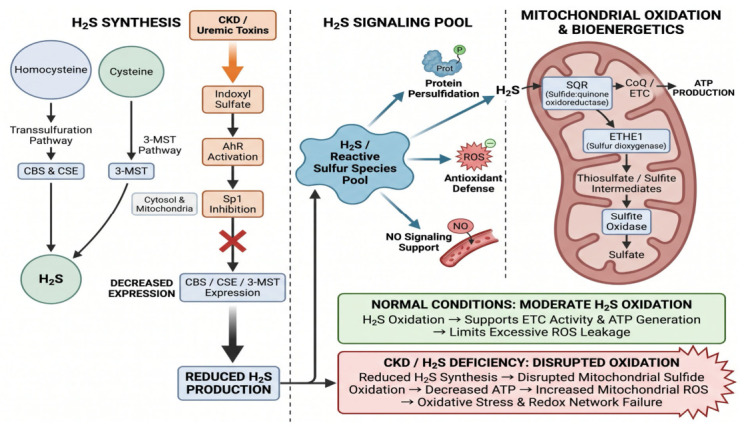
H_2_S biosynthesis, RSS signaling, and mitochondrial sulfide oxidation in CKD redox dysregulation. H_2_S is synthesized from homocysteine and cysteine through CBS- and CSE-dependent transsulfuration and 3-MST-dependent cytosolic and mitochondrial pathways. In CKD, indoxyl sulfate (IS) activates AhR, which suppresses Sp1-dependent transcription of CBS, CSE, and 3-MST, reducing H_2_S production and depleting the H_2_S/RSS pool. Under physiological conditions, H_2_S supports protein persulfidation, antioxidant defense, and NO signaling, while mitochondrial SQR-dependent oxidation donates electrons to the ETC, supporting ATP synthesis and limiting ROS leakage. In CKD, H_2_S deficiency disrupts this oxidation pathway, reducing ATP generation, increasing mitochondrial ROS, and promoting redox network failure. Inhibitory symbols indicate pathways suppressed by uremic toxin signaling; black arrows indicate normal biosynthetic and signaling pathways. Abbreviations: AhR, aryl hydrocarbon receptor; ATP, adenosine triphosphate; CBS, cystathionine β-synthase; CKD, chronic kidney disease; CoQ, coenzyme Q; CSE, cystathionine γ-lyase; ETC, electron transport chain; ETHE1, ethylmalonic encephalopathy protein 1; H_2_S, hydrogen sulfide; 3-MST, 3-mercaptopyruvate sulfurtransferase; NO, nitric oxide; ROS, reactive oxygen species; SQR, sulfide:quinone oxidoreductase; Sp1, specificity protein 1.

Figure 1 integrates the biosynthetic, signaling, and mitochondrial oxidation dimensions of renal H_2_S biology discussed in this section, and illustrates how uremic toxin-driven suppression of H_2_S production disrupts each of these functional axes in CKD.

## 3. H_2_S Deficiency in CKD: Clinical, Experimental, and Metabolic Evidence

### 3.1. Cellular and In Vitro Evidence

Cellular studies indicate that impaired H_2_S biosynthesis develops early in response to metabolic and uremic stress. In renal tubular cells, IS suppresses expression of CBS, CSE, and 3-MST and reduces endogenous H_2_S production through AhR-dependent inhibition of Sp1 transcriptional activity [9]. This effect is accompanied by increased superoxide generation, glutathione depletion, and tubular cell injury, whereas pharmacological AhR inhibition or supplementation with H_2_S donors partially restores antioxidant capacity and cell viability [9]. High-glucose conditions also impair H_2_S-generating pathways. In proximal tubular cells, hyperglycemia promotes ubiquitin–proteasome-mediated degradation of CBS, leading to reduced H_2_S production and increased oxidative stress [32]. Experimental studies further suggest that suppression of H_2_S signaling alters mitochondrial homeostasis and redox regulation. In human tubular cells and murine kidneys, IS induced mitochondrial fragmentation, impaired mitochondrial biogenesis, and disrupted cellular metabolic activity—changes associated with oxidative stress and tubular dysfunction [33]. Together, these findings support a direct mechanistic link between metabolic stress, uremic toxins, impaired H_2_S biosynthesis, and cellular redox imbalance in CKD.

### 3.2. Animal Evidence

Animal models consistently demonstrate reduced H_2_S production capacity in CKD and support impaired biosynthesis, rather than reduced clearance, as a major mechanism underlying H_2_S deficiency. In 5/6-nephrectomized rats, plasma H_2_S concentration, renal and hepatic H_2_S-producing capacity, and renal CBS, CSE, and 3-MST expression were markedly reduced, accompanied by increased oxidative stress and progressive renal functional deterioration [8]. AhR blockade in 5/6-nephrectomized rats restored Sp1 activity, increased renal CBS, CSE, and 3-MST expression, improved glutathione redox balance, and attenuated tubular injury and renal hypoperfusion [34]. The sulfur metabolic disturbance was not confined to the kidney: renal and hepatic H_2_S-producing enzymes were reduced, whereas brain expression was relatively preserved, supporting tissue-specific remodeling of sulfur metabolism during CKD [8].

In diabetic kidney disease models, renal H_2_S changes appear context-dependent. In db/db mice and glucose-exposed renal tubular cells, CBS expression and H_2_S production were reduced, and high glucose promoted ubiquitin–proteasome-mediated CBS degradation, linking hyperglycemia to impaired H_2_S biosynthesis and oxidative renal injury [32]. In an early diabetic kidney disease model, renal H_2_S levels were also reduced despite heterogeneous enzyme changes, including lower CBS, relatively preserved CSE, increased 3-MST, and altered sulfide oxidation pathways [35]. Functional studies support a protective role of H_2_S supplementation in renal injury models. In unilateral ureteral obstruction (UUO) kidneys, reduced H_2_S production occurred together with macrophage infiltration, nuclear factor kappa B (NF-κB) activation, NOD-like receptor family pyrin domain containing 3 (NLRP3) signaling, and fibrosis; NaHS attenuated inflammatory and fibrotic injury [36]. In LPS-induced acute kidney injury (AKI) and ischemia–reperfusion injury models, exogenous H_2_S suppressed oxidative stress, inflammatory cytokine production, NLRP3/caspase-1 activation, and tubular apoptosis [37,38].

### 3.3. Clinical and Metabolic Evidence

Clinical studies consistently demonstrate reduced circulating H_2_S levels across multiple CKD populations. In a cohort of 157 non-dialysis CKD patients, plasma H_2_S concentrations were approximately 48% lower than those of healthy controls (7.3 vs. 14.1 μmol/L) and showed a positive correlation with estimated glomerular filtration rate (eGFR) (ρ = 0.577), indicating progressive decline with worsening renal function [39]. In maintenance hemodialysis patients, plasma H_2_S and sulfhemoglobin levels were reduced, whereas homocysteine and cysteine concentrations were elevated, accompanied by downregulation of CSE expression in peripheral blood cells [40]. These findings indicate that disruption of sulfur-mediated redox regulation persists across advanced CKD and dialysis populations rather than being confined to early-stage disease.

Metabolic studies further demonstrate coordinated disruption of the transsulfuration pathway in CKD. Under physiological conditions, homocysteine is converted to cysteine through CBS- and CSE-mediated reactions that also generate H_2_S. A remnant kidney study showed marked downregulation of CBS, CSE, and 3-MST expression in renal tissue, together with reduced hepatic CBS and CSE expression, indicating systemic loss of H_2_S-producing capacity during CKD progression [8]. CKD is also associated with accumulation of sulfur metabolites linked to impaired transsulfuration. In hemodialysis patients, lanthionine and homolanthionine concentrations increased by up to two orders of magnitude in parallel with low plasma H_2_S and hyperhomocysteinemia [41]. Experimental studies further demonstrated that lanthionine interferes with CBS-dependent sulfur metabolism and disrupts transsulfuration pathway activity, suggesting a potential feed-forward mechanism contributing to persistent H_2_S deficiency in uremia [42]. Together, these findings indicate that CKD is accompanied by coordinated disruption of sulfur amino acid metabolism extending beyond a single H_2_S-producing pathway.

### 3.4. Conceptual Integration: H_2_S-Deficient, Toxin-Driven Redox State

Clinical, experimental, and metabolic evidence collectively support the concept that CKD represents an H_2_S-deficient state. Reduced circulating H_2_S levels, downregulation of CBS, CSE, and 3-MST, and impaired transsulfuration collectively indicate coordinated disruption of H_2_S homeostasis rather than an isolated biochemical abnormality [34,39]. These abnormalities are observed across renal tubular cells, animal CKD models, and dialysis populations, suggesting that impaired sulfur-mediated redox regulation develops throughout CKD progression.

Uremic toxins, particularly IS, appear to play a central role in this process by suppressing H_2_S biosynthesis through AhR-dependent inhibition of Sp1 activity [9]. Reduced H_2_S availability is accompanied by oxidative stress, inflammatory signaling, mitochondrial dysfunction, and tubular injury, whereas restoration of H_2_S-producing pathways or H_2_S supplementation partially improves these abnormalities [9,34]. It should be noted, however, that whether H_2_S deficiency functions as a primary upstream driver or primarily as a downstream marker of broader uremic and oxidative stress remains to be directly established. Most supporting evidence is derived from experimental models, and direct causal evidence in human CKD tissues is limited. The proposed relationship between H_2_S deficiency and coordinated redox dysregulation should therefore be interpreted as a mechanistically supported framework rather than an established causal conclusion.

Beyond this causal uncertainty, the pathological significance of H_2_S deficiency may not be uniform across all CKD contexts. H_2_S exerts concentration-dependent biphasic effects on mitochondrial function: while low-to-moderate concentrations support oxidative phosphorylation and confer cytoprotection, excessive sulfide inhibits cytochrome c oxidase and promotes oxidative injury, as demonstrated in studies using mitochondria-targeted H_2_S donors [43]. This biphasic profile implies that the consequences of H_2_S depletion depend in part on the prevailing mitochondrial and metabolic state. Furthermore, H_2_S system responses are not uniformly suppressive across CKD subtypes or stages. In early diabetic kidney disease models, 3-MST expression was paradoxically increased despite overall reductions in renal H_2_S levels, suggesting stage- and context-specific remodeling of sulfur metabolism rather than a simple linear decline [35]. In the physiologically hypoxic renal medulla, H_2_S functions as an oxygen-sensitive signaling molecule, and context-dependent regulation of its synthesis may carry adaptive significance under conditions of altered oxygen tension [18]. Whether such responses represent transient compensatory adjustments or contribute to sustained redox dysregulation remains to be determined. Taken together, while existing evidence predominantly supports H_2_S deficiency as a pathological rather than compensatory phenomenon in established CKD, its functional significance should be interpreted in light of disease stage, oxygen tension, mitochondrial status, and sulfur substrate availability. These considerations reinforce the need for stage-specific and mechanistically informed approaches when evaluating H_2_S as a therapeutic target in CKD.

A further dimension of complexity is that CKD is not a homogeneous condition, and the pattern of sulfur metabolic disruption varies across disease stages, etiologies, and treatment modalities. In non-dialysis CKD, plasma H_2_S declines progressively with eGFR and correlates positively with residual renal function, while IS accumulates in a reciprocal fashion from stage III onward, suggesting that biosynthetic suppression and toxin-driven enzyme inhibition intensify in parallel as renal function deteriorates [39,44]. In diabetic kidney disease, the mechanism of H_2_S depletion differs from that in non-diabetic CKD: hyperglycemia promotes ubiquitin–proteasome-mediated CBS degradation and increases renal SQR expression, indicating that oxidative clearance as well as biosynthetic suppression contributes to H_2_S deficiency in this context [35]. In maintenance hemodialysis populations, IS and p-cresyl sulfate (pCS) reach their highest circulating concentrations, yet high-volume hemodiafiltration achieves only modest and transient reductions in pre-dialysis IS levels, reflecting the dominant contribution of ongoing gut production and protein binding to toxin burden rather than dialytic clearance alone [44,45]. Across modalities, kidney transplantation normalizes IS concentrations toward control levels, whereas peritoneal dialysis and hemodialysis leave them substantially elevated [46]. These stage- and modality-specific differences suggest that the relative contributions of impaired biosynthesis, toxin-driven enzyme suppression, and altered sulfide oxidation to sulfur metabolic disruption may vary across the CKD continuum, with implications for the timing and targeting of H_2_S-based therapeutic strategies. These abnormalities establish the mechanistic context for the uremic toxin-driven suppression of H_2_S biosynthesis examined in the following section.

## 4. Uremic Toxin-Driven Suppression of H_2_S in CKD

### 4.1. Uremic Toxin Accumulation and Sustained AhR Activation

CKD is characterized by progressive accumulation of uremic toxins due to impaired renal clearance and altered tubular secretion. Protein-bound uremic toxins are of particular relevance because they are poorly removed by conventional dialysis. IS is one of the most extensively studied toxins and is strongly associated with renal and cardiovascular injury, with circulating levels increasing markedly as renal function declines [47,48]. In advanced CKD and ESRD, plasma IS concentrations can rise up to ~100-fold compared with those in individuals with normal renal function, reflecting both reduced excretion and ongoing production [49,50]. Its high protein-binding capacity (>90% bound to albumin) further limits dialytic removal and promotes systemic retention [51].

IS is generated through the gut–liver–kidney axis. Dietary tryptophan is metabolized by intestinal microbiota into indole, which is subsequently converted to IS in the liver before entering the circulation [51]. Under physiological conditions, IS is actively secreted by proximal tubular cells via organic anion transporters, whereas in CKD, reduced tubular function and transporter activity lead to progressive accumulation in the circulation and tissues [52]. Beyond its retention, IS induces oxidative stress, inflammatory signaling, mitochondrial dysfunction, and fibrosis, thereby contributing to CKD progression [53,54].

IS also functions as an endogenous ligand of the AhR, linking toxin accumulation to transcriptional reprogramming. Upon activation, AhR translocates to the nucleus, heterodimerizes with the aryl hydrocarbon receptor nuclear translocator (ARNT), and regulates target genes such as cytochrome P450 family 1 subfamily A member 1 (CYP1A1) and aryl hydrocarbon receptor repressor (AHRR) [55,56]. In addition to canonical detoxification pathways, AhR interacts with signaling networks including NF-κB, activator protein 1 (AP-1), and mitogen-activated protein kinase (MAPK) cascades, extending its role to inflammation, oxidative stress, and cellular stress responses [57,58]. Sustained toxin exposure results in persistent AhR activation across renal, vascular, and immune compartments, establishing a chronic signaling state that promotes oxidative stress and inflammation [56,58].

While IS is the most extensively studied uremic toxin in the context of H_2_S suppression, CKD is characterized by simultaneous accumulation of multiple AhR-activating toxins that may collectively amplify redox network disruption beyond what IS alone produces. Indole-3-acetic acid (IAA), another tryptophan-derived gut microbial metabolite, activates AhR through p38 MAPK and NF-κB-dependent pathways in endothelial cells, induces COX-2-mediated oxidative stress, and independently predicts cardiovascular mortality in CKD populations [59]. Kynurenine-pathway metabolites generated through host tryptophan catabolism similarly activate AhR and suppress Wnt/β-catenin signaling, contributing to impaired angiogenesis and CKD progression through mechanisms that converge with IS-driven AhR activation [60]. pCS, derived from gut microbial tyrosine metabolism, promotes renal tubular oxidative stress and fibrosis through ROS-dependent pathways and epigenetic suppression of Klotho, and among protein-bound toxins shows the strongest independent association with cardiovascular events after mutual adjustment with IS [61]. These toxins accumulate in parallel as eGFR declines and share the capacity to activate AhR, generate endothelial ROS, and suppress antioxidant defenses, suggesting that the net burden of AhR-driven H_2_S suppression and redox network disruption in CKD reflects a multi-toxin network effect rather than the action of IS in isolation.

### 4.2. The IS–AhR–Sp1 Axis: A Mechanistic Link to H_2_S Deficiency

Sp1 is a key transcription factor that maintains basal expression of H_2_S-producing enzymes. It binds GC-rich promoter regions of CBS and CSE and supports transcriptional activity in renal tubular and endothelial cells [62,63,64]. Loss of Sp1 activity reduces expression of these enzymes and limits H_2_S production [65].

In CKD, IS-driven activation of AhR disrupts this transcriptional program by suppressing Sp1-dependent gene expression. In LLC-PK1 proximal tubular cells, exposure to IS markedly reduced endogenous H_2_S release, accompanied by downregulation of CBS, CSE, and 3-MST at both the mRNA and protein levels [9]. Functional inhibition of endogenous H_2_S synthesis using aminooxyacetic acid further aggravated IS-induced tubular injury, whereas supplementation with H_2_S donors including NaHS and GYY4137 attenuated lactate dehydrogenase release and improved cell viability, supporting a protective role of endogenous H_2_S against uremic toxin-mediated tubular damage. Mechanistically, IS significantly reduced Sp1 DNA-binding activity without substantially altering total Sp1 protein expression, indicating functional impairment of Sp1-dependent transcription rather than transcription factor depletion [9]. Importantly, pharmacological blockade of AhR with CH-223191 restored Sp1 activity and rescued expression of H_2_S-producing enzymes, identifying AhR-dependent suppression of Sp1 as a direct upstream mechanism linking IS exposure to impaired H_2_S biosynthesis [9]. IS-mediated suppression of H_2_S formation was also associated with increased superoxide production and depletion of intracellular glutathione, whereas exogenous H_2_S supplementation partially restored antioxidant capacity and attenuated tubular oxidative injury [9].

**Figure 2:**
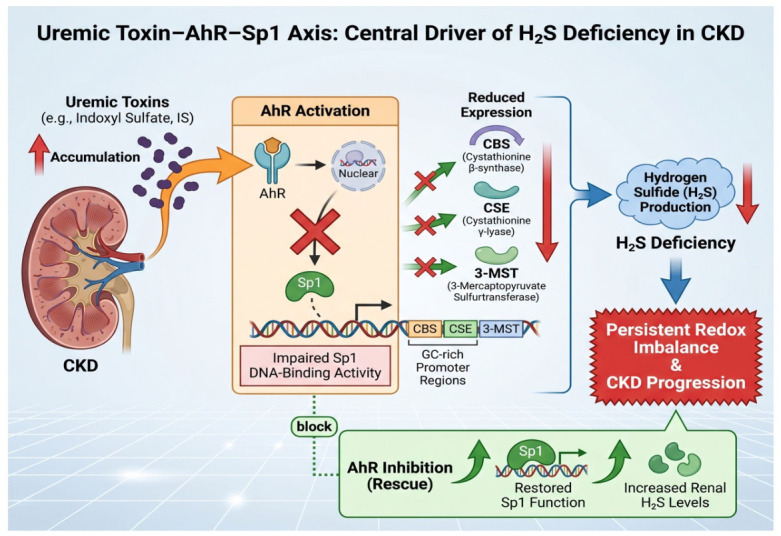
Uremic toxin–AhR–Sp1 axis in H_2_S deficiency in CKD. In CKD, indoxyl sulfate (IS) activates the aryl hydrocarbon receptor (AhR), which suppresses specificity protein 1 (Sp1)-dependent transcription of hydrogen sulfide (H_2_S)-producing enzymes, including cystathionine β-synthase (CBS), cystathionine γ-lyase (CSE), and 3-mercaptopyruvate sulfurtransferase (3-MST). This suppresses H_2_S biosynthesis and disrupts redox network integration. AhR inhibition restores Sp1 activity and H_2_S signaling. Abbreviations: AhR, aryl hydrocarbon receptor; CBS, cystathionine β-synthase; CSE, cystathionine γ-lyase; CKD, chronic kidney disease; H_2_S, hydrogen sulfide; IS, indoxyl sulfate; Sp1, specificity protein 1; 3-MST, 3-mercaptopyruvate sulfurtransferase.

Similar findings were observed in vivo in 5/6-nephrectomized rats, where CKD was associated with accumulation of IS and homocysteine, reduced renal H_2_S levels, and marked downregulation of CBS, CSE, and 3-MST expression in remnant kidneys [34]. These changes were accompanied by reduced Sp1 protein expression and DNA-binding activity, increased superoxide generation, glutathione depletion, impaired renal perfusion, and tubular injury [34]. Chronic AhR inhibition with CH-223191 restored Sp1 activity, increased expression of H_2_S-producing enzymes, normalized renal H_2_S levels, improved glutathione redox balance, and partially reversed renal functional impairment and cortical microvascular hypoperfusion [34]. Notably, AhR blockade also reduced homocysteine accumulation, suggesting that suppression of the transsulfuration pathway contributes to the H_2_S-deficient phenotype observed in CKD [34]. Collectively, these findings support a mechanistic model in which IS-driven AhR activation contributes to suppression of Sp1-dependent H_2_S biosynthesis and disruption of sulfur-mediated redox regulation in CKD. Figure 2 illustrates AhR activation, Sp1 inhibition, and reduced transcription of H_2_S-producing enzymes.

Beyond the kidney, IS–AhR signaling mediates redox dysregulation through mechanisms that are largely independent of Sp1-dependent H_2_S suppression. In vascular endothelial cells, IS activates AhR and induces downstream CYP1A1- and NADPH oxidase (NOX)-related pathways, leading to increased ROS production, nitrotyrosine formation, reduced endothelial nitric oxide synthase (eNOS) expression, and decreased NO bioavailability [47,66]. In rat aortic rings, IS exposure impaired endothelium-dependent vasorelaxation, whereas AhR inhibition partially restored endothelial responsiveness and reduced oxidative stress signaling [66]. These alterations promote endothelial dysfunction and impair vascular homeostasis in CKD.

In cardiac tissue, IS-induced AhR activation similarly promotes oxidative stress through CYP1-mediated pathways, leading to increased ROS production and cardiomyocyte hypertrophy [67]. Experimental studies further demonstrate associated mitochondrial dysfunction and redox imbalance in uremic cardiomyopathy models [67]. Together, these findings suggest that persistent AhR activation functions as a broad cross-tissue regulator of oxidative stress signaling in CKD.

Collectively, the IS–AhR pathway appears to function as an important upstream regulator of redox dysregulation in CKD. In renal tubular cells, this effect is mediated primarily through Sp1-dependent suppression of H_2_S biosynthesis, whereas in vascular and cardiac tissues, AhR signaling promotes oxidative stress through CYP1- and NOX-dependent mechanisms that extend beyond the canonical IS–AhR–Sp1–H_2_S axis (Table 1).

**Table 1:** Tissue-specific effects of indoxyl sulfate (IS)–aryl hydrocarbon receptor (AhR) signaling on redox dysregulation and organ injury in CKD.

Biological Context	Mechanistic Axis	Impact on H_2_S Homeostasis and Redox Signaling	Pathophysiological Consequence	References
Renal tubular cells	IS activates AhR, suppressing Sp1-dependent transcription of CBS, CSE, and 3-MST	Decreased H_2_S bioavailability; glutathione depletion; increased oxidative stress	Tubular injury and progression of tubulointerstitial fibrosis	[9]
Animal models (5/6-nephrectomy)	AhR activation impairs transsulfuration pathway flux and reduces Sp1 DNA-binding activity	Reduced renal H_2_S levels; disrupted sulfide oxidation and redox signaling	Progressive renal dysfunction and imbalance of mitochondrial redox regulation	[34]
Vascular endothelium	IS-induced AhR activation upregulates CYP1A1 and NOX, promoting endothelial oxidative stress	Reduced NO bioavailability; eNOS uncoupling; disrupted endothelial redox balance	Endothelial dysfunction, vascular injury, and impaired vasorelaxation	[47,66]
Cardiomyocytes	IS–AhR signaling activates CYP1-mediated ROS generation and mitochondrial oxidative pathways	Oxidative stress amplification; impaired mitochondrial bioenergetics	Cardiac hypertrophy and uremic cardiomyopathy	[67]

Abbreviations: IS, indoxyl sulfate; AhR, aryl hydrocarbon receptor; Sp1, specificity protein 1; H_2_S, hydrogen sulfide; CBS, cystathionine β-synthase; CSE, cystathionine γ-lyase; 3-MST, 3-mercaptopyruvate sulfurtransferase; CYP1A1, cytochrome P450 1A1; NOX, NADPH oxidase; ROS, reactive oxygen species; NO, nitric oxide; eNOS, endothelial nitric oxide synthase.

### 4.3. Integration with Inflammatory and Metabolic Stress Pathways

The IS–AhR–Sp1 axis does not function in isolation but interacts closely with inflammatory and metabolic stress pathways that are persistently activated in CKD. Experimental studies have shown that reduced H_2_S availability amplifies inflammatory signaling through NF-κB- and NLRP3-dependent mechanisms. In UUO kidneys, reduced endogenous H_2_S production was accompanied by increased NF-κB activation, enhanced NLRP3 inflammasome signaling, and marked M1/M2 macrophage infiltration, together with progressive renal fibrosis [36]. Administration of NaHS suppressed NF-κB and NLRP3 activation, reduced macrophage accumulation, and attenuated tubulointerstitial injury and fibrosis [36]. Similar findings were observed in lipopolysaccharide-induced AKI, where loss of endogenous H_2_S and reduced 3-MST expression occurred together with increased Toll-like receptor 4 (TLR4), NLRP3, caspase-1, IL-1β, and tumor necrosis factor-alpha (TNF-α) expression, while NaHS treatment improved renal injury and suppressed inflammatory signaling [37]. In renal ischemia–reperfusion models, exogenous H_2_S also reduced NLRP3/apoptosis-associated speck-like protein containing a CARD (ASC)/caspase-1 activation and limited cytokine release and tubular apoptosis through Nrf2-dependent mechanisms [38,68]. These studies indicate that impaired H_2_S signaling not only weakens antioxidant defense but also facilitates inflammasome priming and sustained cytokine activation during renal injury.

Inflammatory stress further intersects with metabolic dysregulation and uremic toxin signaling in CKD. Hyperhomocysteinemia and impaired transsulfuration flux reduce substrate availability for H_2_S synthesis, whereas hyperglycemia and oxidative stress suppress CBS and CSE expression. In parallel, IS activates AhR-dependent inflammatory pathways in macrophages and endothelial cells. In THP-1-derived macrophages, IS increased pro-IL-1β transcription together with activation of AhR, NF-κB p65, and MAPK signaling cascades, although full NLRP3 inflammasome activation was not observed [69]. In vascular endothelial cells, AhR activation mediated IS-induced leukocyte adhesion and E-selectin expression through AP-1-dependent transcriptional signaling [70]. Experimental studies also showed that H_2_S donors suppress monocyte chemoattractant protein-1 (MCP-1), TNF-α, IL-1β, and IL-6 production through inhibition of NF-κB signaling in macrophages and chronic renal injury models [28,71].

These inflammatory interactions acquire deeper mechanistic significance when considered within an immunometabolic framework. Macrophage polarization is tightly coupled to cellular redox and metabolic state: M1 activation is supported by glycolytic reprogramming and mitochondrial ROS generation through the ROS–ataxia telangiectasia mutated kinase (ATM)–checkpoint kinase 2 (Chk2)–pyruvate kinase M2 (PKM2) axis, whereas M2 polarization depends on oxidative phosphorylation and fatty acid oxidation [72]. H_2_S directly modulates this balance—in myocardial infarction models, NaHS promotes M2 polarization by enhancing mitochondrial biogenesis and fatty acid oxidation, reducing inflammatory remodeling [73]. At the molecular level, widespread protein persulfidation in activated macrophages inhibits NLRP3 inflammasome assembly and protects against oxidative-inflammatory cell death, suggesting that H_2_S deficiency in CKD may lower the threshold for inflammasome activation by reducing this endogenous persulfidation-dependent brake [74]. In the uremic context, IS drives macrophages toward chronic M1-like states at high concentrations through AhR–NOX–ROS pathways, while at moderate CKD-stage concentrations it may instead promote mixed profibrotic M2-like phenotypes with concurrent pro-inflammatory cytokine secretion—a pattern consistent with the immune reprogramming observed in CKD-associated inflammaging [75]. Collectively, these findings suggest that H_2_S deficiency and IS accumulation cooperatively dysregulate macrophage immunometabolism in CKD, shifting innate immune cells toward self-sustaining inflammatory states that extend the impact of redox network failure beyond tubular and vascular compartments.

### 4.4. Gut Microbiota and Dysbiosis-Driven Modulation of Sulfur Metabolism in CKD

The gut microbiota represents an important but underappreciated contributor to systemic sulfur homeostasis. Intestinal H_2_S is generated through two principal microbial routes: dissimilatory sulfate reduction by sulfate-reducing bacteria such as *Desulfovibrio*, and cysteine-based amino acid degradation pathways that are now recognized as quantitatively more important and phylogenetically widespread across the gut microbiome [76,77]. The capacity for cysteine-derived H_2_S production is present in essentially all individuals and exceeds that of classical sulfate reduction under physiological dietary conditions [77].

In CKD, this microbiota-dependent sulfur network intersects with uremic toxin biology in mechanistically relevant ways. In a murine CKD model, a high sulfur amino acid diet enhanced microbial protein S-sulfhydration, suppressed bacterial tryptophanase activity, reduced IS production, and attenuated CKD progression—demonstrating that dietary sulfur intake can modulate the gut–liver–kidney toxin axis through post-translational modification of microbial enzymes [78]. Conversely, gut microbiota also reinforce host antioxidant capacity through generation of RSS that support epithelial redox defense [79]. In human CKD, however, circulating H_2_S is reduced and lanthionine elevated despite the presence of sulfidogenic taxa, suggesting that systemic sulfur metabolite changes in CKD reflect host metabolic dysfunction rather than increased microbial H_2_S overproduction [80]. Collectively, these findings indicate that CKD-associated dysbiosis may disrupt the normal balance between microbiota-derived sulfur species and host sulfur metabolism, contributing to systemic RSS depletion through mechanisms that extend beyond impaired endogenous biosynthesis.

## 5. H_2_S in Redox Network Integration

### 5.1. ROS–NO–H_2_S Interactions

H_2_S participates in vascular redox regulation primarily through interactions with NO and ROS signaling rather than direct free radical scavenging. In endothelial cells, H_2_S increases NO bioavailability by enhancing eNOS activity through multiple mechanisms, including Akt-dependent phosphorylation, stabilization of eNOS dimers, and protein persulfidation [81]. In endothelial-specific experiments, H_2_S-induced vasorelaxation and angiogenesis were markedly attenuated in eNOS-deficient mice, indicating functional dependence of H_2_S signaling on intact NO pathways [82]. H_2_S also promotes eNOS protein stability through microRNA-455-3p-dependent regulation, resulting in sustained NO production and improved endothelial signaling under oxidative stress conditions [83]. In parallel, chemical interactions between H_2_S and NO generate intermediates such as thionitrous acid and nitrosopersulfide, which function as reactive sulfur–nitrogen signaling species capable of modulating NO-responsive soluble guanylate cyclase signaling [84]. These findings support the concept that H_2_S and NO function as interdependent signaling systems within vascular redox regulation.

Excess ROS disrupts this coordinated signaling network. Increased oxidative stress promotes eNOS uncoupling, reduces NO bioavailability, and shifts endothelial signaling toward vasoconstrictive and proinflammatory states. Experimental studies further show that reduced H_2_S availability is accompanied by glutathione depletion, increased superoxide generation, and impaired endothelial relaxation in CKD-related and hyperglycemic models [9,85]. In hyperglycemic vascular tissue, the H_2_S donor AP123 restored eNOS phosphorylation, improved endothelium-dependent vasorelaxation, and reduced oxidative stress markers through cAMP response element-binding protein (CREB)-dependent signaling pathways [85]. These observations indicate that ROS, NO, and H_2_S operate as interconnected signaling systems that collectively regulate endothelial homeostasis and vascular redox balance.

### 5.2. Protein Persulfidation and Redox Signaling

Protein persulfidation is a major downstream mechanism through which H_2_S regulates redox-sensitive signaling pathways. This reversible post-translational modification converts reactive cysteine thiols into persulfides and alters protein activity, protein–protein interactions, and susceptibility to oxidative injury. Experimental studies indicate that persulfidation preferentially targets oxidized cysteine residues and may protect proteins from irreversible overoxidation during oxidative stress [86]. One of the best-characterized targets is Kelch-like ECH-associated protein 1 (Keap1), a suppressor of Nrf2 signaling. In experimental injury models, H_2_S-induced Keap1 persulfidation disrupted the Keap1–Nrf2 interaction and promoted Nrf2-dependent antioxidant gene transcription, thereby increasing cellular resistance to oxidative stress [86]. These findings support persulfidation as a direct molecular link between H_2_S signaling and endogenous antioxidant defense systems.

Persulfidation also regulates proteins involved in endothelial signaling, mitochondrial metabolism, and cellular stress adaptation. In endothelial cells, H_2_S induces persulfidation of eNOS at Cys443 and enhances Akt-dependent eNOS activation, leading to increased NO production and improved vasodilatory signaling [81]. Experimental studies further demonstrate that H_2_S-mediated persulfidation modulates mitochondrial and metabolic pathways. ATP synthase persulfidation increases mitochondrial respiratory activity under low-concentration H_2_S exposure, whereas glyceraldehyde-3-phosphate dehydrogenase (GAPDH) persulfidation alters glycolytic signaling and redirects cellular metabolism toward antioxidant NADPH-generating pathways [11]. Persulfidation-dependent regulation of autophagy and stress-response pathways has also been reported in multiple experimental systems [87]. Reduced H_2_S availability during CKD progression may therefore impair several persulfidation-dependent pathways involved in antioxidant defense, endothelial homeostasis, mitochondrial bioenergetics, and metabolic adaptation.

An emerging and mechanistically significant extension of persulfidation biology concerns epigenetic regulatory proteins. H_2_S inhibits histone deacetylase (HDAC) activity through persulfidation of reactive cysteine residues within the HDAC catalytic domain, shifting chromatin toward a more acetylated, transcriptionally permissive state—an effect demonstrated in experimental neurodegeneration models and potentially relevant to renal gene regulation [88,89]. This HDAC-suppressive mechanism intersects with the AhR signaling axis in CKD: AhR recruits coactivator complexes that alter local histone acetylation and methylation at target gene promoters, and HDAC inhibition can in turn suppress AhR expression, suggesting a reciprocal chromatin-level feedback between uremic toxin signaling and sulfur-mediated epigenetic control [90]. A directly CKD-relevant example is provided by renal ischemia–reperfusion injury, in which CSE promoter hypermethylation reduces CSE expression and H_2_S bioavailability; pharmacological demethylation of the CSE promoter restores H_2_S production and attenuates oxidative renal injury, demonstrating that epigenetic silencing of H_2_S-producing enzymes is a functionally significant mechanism of H_2_S deficiency beyond transcriptional suppression via the IS–AhR–Sp1 axis [91]. Collectively, these findings suggest that H_2_S deficiency in CKD may be self-reinforcing at the epigenetic level: reduced persulfidation capacity permits HDAC overactivation and promoter hypermethylation, further suppressing CBS and CSE expression and perpetuating sulfur metabolic disruption through chromatin-level mechanisms.

### 5.3. RSS Signaling Network: Polysulfides, Persulfides, and Thiosulfate as Bioactive Mediators

Although H_2_S is the most studied RSS, its downstream oxidation products—cysteine persulfides (CysSSH), GSSH, and polysulfides—may represent the principal bioactive mediators of sulfur-dependent redox signaling. These species are generated via SQR-dependent H_2_S oxidation and are present at concentrations exceeding 100 μM in mammalian cells, exhibiting greater nucleophilicity and reducing capacity than their thiol counterparts [92,93]. Polysulfides can directly transfer sulfane sulfur to reactive cysteine residues, and many effects previously attributed to H_2_S are now understood to be mediated by these more oxidized intermediates [94]. Key downstream targets include Keap1, whose persulfidation disrupts Nrf2 suppression and activates antioxidant gene transcription, and metabolic enzymes such as GAPDH and ATP synthase, whose persulfidation redirects metabolism toward NADPH generation and supports mitochondrial bioenergetics [93,95]. In CKD, coordinated depletion across these interconnected RSS pools—including the bound sulfane sulfur reservoir and thiosulfate as a terminal sulfur oxidation product—may impair redox buffering capacity beyond what H_2_S measurement alone reflects, underscoring the need to consider the broader RSS network when interpreting sulfur-mediated redox dysregulation in this disease.

### 5.4. Mitochondrial Integration and Network-Level Redox Control

H_2_S links mitochondrial respiration with redox regulation in a concentration-dependent manner. At physiological concentrations, H_2_S functions as an electron donor to the electron transport chain through SQR, supporting oxidative phosphorylation and ATP generation. In endothelial cells, GYY4137-derived H_2_S increased mitochondrial oxygen consumption and transferred electrons into the electron transport chain through SQR-dependent mechanisms, whereas pharmacological SQR inhibition abolished these effects [96]. Experimental studies further demonstrated that intramitochondrial H_2_S production by 3-MST maintained electron transport and cellular bioenergetics under basal conditions [6]. Low concentrations of mitochondria-targeted H_2_S donors also increased ATP production, preserved mitochondrial membrane potential, and reduced oxidative injury in stressed endothelial cells [97]. These findings indicate that physiological H_2_S supports mitochondrial respiration while limiting excessive ROS accumulation during electron transport.

At higher concentrations, H_2_S exerts opposite effects on mitochondrial function. Excess H_2_S inhibits cytochrome c oxidase (Complex IV), disrupts oxidative phosphorylation, and promotes ROS generation and metabolic stress [98]. In colon cells, high H_2_S exposure impaired mitochondrial bioenergetics, increased reverse electron transport, altered NAD^+^/NADH and CoQ/CoQH2 balance, and induced broader metabolic reprogramming through SQR-dependent pathways [99]. Experimental studies further showed that SQR-dependent H_2_S oxidation regulates mitochondrial redox balance by coupling sulfide metabolism to electron transport and mitochondrial redox cycling pathways [99].

## 6. Redox Triad Failure in CKD: A Network Model of Disease Progression

### 6.1. Conceptual Framework: Loss of Coordinated Redox Regulation

In CKD, redox imbalance reflects disruption of interconnected signaling systems rather than isolated excess of ROS. ROS, NO, and sulfur-based redox pathways are concurrently altered, and impaired crosstalk among these systems disrupts cellular signaling, mitochondrial function, vascular tone, and metabolic regulation [2,100,101]. Physiological levels of ROS and NO are required for normal signaling, and both deficiency and excess are detrimental, underscoring the importance of coordinated redox regulation rather than global suppression [102].

Redox triad failure therefore denotes loss of coordinated regulation among ROS, NO, and reactive sulfur systems [24,101]. From a systems-biology perspective, these three axes do not operate as parallel but independent pathways; rather, they form a coupled regulatory network governed by mutual feedback interactions. Under physiological conditions, H_2_S enhances NO bioavailability through eNOS activation and protein persulfidation, while NO and H_2_S together limit ROS accumulation via Nrf2-dependent antioxidant gene transcription [5,81,101]. This network maintains redox homeostasis through dynamic, reciprocal regulation rather than simple additive effects.

In CKD, this coupled network is destabilized by feed-forward amplification. Uremic toxin-driven AhR activation suppresses H_2_S biosynthesis, which in turn reduces glutathione availability and Nrf2 activity, permitting further ROS accumulation [9,34]. Excess ROS promotes eNOS uncoupling, shifting NO synthase from NO generation toward superoxide production and further depleting NO bioavailability [102]. Reduced NO signaling impairs the cooperative H_2_S–NO axis, diminishing the capacity for persulfidation-dependent Nrf2 activation and amplifying oxidative injury [81,101]. These interactions constitute a self-reinforcing cycle in which loss of any single axis propagates dysfunction across the network, a property consistent with the positive feedback architecture described in redox systems-biology models.

**Figure 3:**
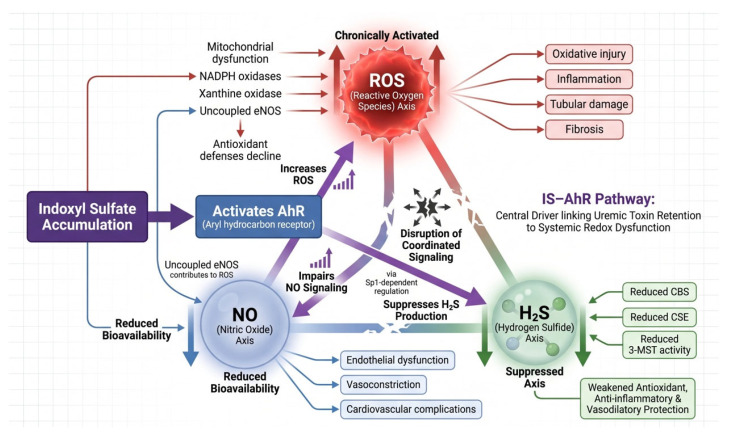
Redox triad failure in CKD: disruption of coordinated ROS–NO–H_2_S signaling. In CKD, redox imbalance reflects disruption of coordinated regulation among reactive oxygen species (ROS), nitric oxide (NO), and H_2_S pathways. Uremic toxin-mediated activation of AhR increases ROS generation, impairs NO bioavailability, and suppresses H_2_S production through Sp1-dependent mechanisms. This network disruption promotes mitochondrial dysfunction, endothelial impairment, inflammation, and fibrosis, linking redox dysregulation to progressive renal and cardiovascular injury. Abbreviations: AhR, aryl hydrocarbon receptor; CKD, chronic kidney disease; eNOS, endothelial nitric oxide synthase; H_2_S, hydrogen sulfide; IS, indoxyl sulfate; NO, nitric oxide; ROS, reactive oxygen species.

A critical feature of such coupled networks is the existence of threshold behavior. Under conditions of moderate oxidative stress, Nrf2-dependent compensatory responses can maintain redox balance within an adaptive range. However, experimental and clinical evidence indicates that Nrf2 activity is progressively suppressed in advanced CKD, associated with Keap1 upregulation and sustained NF-κB activation, suggesting that the compensatory capacity of the antioxidant network diminishes as disease progresses [103]. Computational modeling of redox networks further demonstrates that mutual inhibition between glutathione and ROS can generate bistability, in which the system transitions from a low-ROS adaptive state to a high-ROS self-sustaining state once a critical threshold is crossed [104]. In the context of CKD, progressive H_2_S depletion, eNOS uncoupling, and Nrf2 repression may collectively represent the molecular basis for such a tipping point—a transition beyond which redox dysregulation becomes self-perpetuating and less amenable to single-target interventions. Figure 3 illustrates the coordinated disruption of ROS activation, NO impairment, and H_2_S suppression within this network.

### 6.2. Coordinated Dysregulation of ROS, NO, and H_2_S Axes

Persistent activation of the ROS axis is a central feature of CKD and is driven by multiple enzymatic and mitochondrial sources. Experimental studies have shown that mitochondrial dysfunction and NOX activation markedly increase ROS production in renal tubular and vascular cells, leading to oxidative injury and fibrotic remodeling. In diabetic kidney disease models, NOX4 overexpression increased mitochondrial ROS generation, DNA damage, and tubulointerstitial fibrosis, whereas genetic or pharmacological NOX inhibition attenuated oxidative injury and renal fibrosis [105]. In remnant kidney models, impaired antioxidant defense was accompanied by reduced Nrf2 activity and depletion of endogenous antioxidant systems, contributing to sustained oxidative stress and progressive renal injury [8]. Experimental CKD studies further demonstrated increased lipid peroxidation, protein oxidation, and inflammatory activation together with mitochondrial structural abnormalities, indicating that excessive ROS production extends beyond isolated oxidant accumulation and disrupts broader cellular signaling networks [106].

The NO axis is concurrently impaired through reduced NO bioavailability and endothelial dysfunction. In experimental endothelial models, IS reduced eNOS expression, increased superoxide generation, and impaired endothelium-dependent vasorelaxation, together with increased NOX4 expression and nitrotyrosine formation [47]. Subsequent studies further suggested that AhR activation contributes to IS-mediated endothelial oxidative stress and vascular dysfunction [70,107]. Oxidative stress additionally promotes eNOS uncoupling, shifting eNOS from NO generation toward superoxide production and amplifying vascular oxidative stress. In isolated vascular tissue, IS exposure increased nitrotyrosine formation and markedly reduced acetylcholine-mediated endothelial relaxation, indicating direct disruption of NO-dependent vascular signaling [47]. Similar abnormalities have been observed in CKD and hyperhomocysteinemia models, where impaired eNOS activity was accompanied by endothelial dysfunction, vascular stiffness, and reduced renovascular relaxation [108]. These findings support a mechanistic link between oxidative stress, impaired NO signaling, and progressive vascular injury in CKD.

The sulfur axis, represented by H_2_S, is also diminished during CKD progression. In 5/6-nephrectomized rats, plasma H_2_S levels, together with renal and hepatic H_2_S-producing capacity, were markedly reduced, accompanied by downregulation of CBS, CSE, and 3-MST expression [8]. Experimental studies further showed that IS suppresses H_2_S biosynthesis through AhR-dependent inhibition of Sp1 activity, resulting in glutathione depletion, increased superoxide generation, and tubular injury [9]. Importantly, mitochondrial dysfunction and NOX-dependent ROS generation can sustain oxidative injury and renal fibrosis independently of H_2_S status, as demonstrated in NOX4 overexpression and fatty acid oxidation-deficient models [105,109]. H_2_S deficiency may therefore amplify rather than initiate redox dysregulation, and the relative contribution of each axis is likely to vary across CKD etiology, disease stage, and tissue compartment.

### 6.3. Network Amplification: The IS–AhR Axis as an Upstream Driver

Redox triad failure in CKD is sustained by feed-forward interactions within this network. As renal function declines, IS accumulates and activates the AhR, positioning uremic toxins as upstream drivers of redox disruption [55]. AhR activation enhances ROS generation and inflammatory signaling, reinforcing oxidative stress and tissue injury [58].

The IS–AhR axis directly suppresses the sulfur pathway by downregulating CBS, CSE, and 3-MST through inhibition of Sp1 activity, leading to reduced H_2_S production [9]. At the same time, AhR signaling promotes ROS generation and impairs NO signaling, thereby destabilizing all three components of the redox system [47,56]. Beyond the kidney, IS–AhR signaling reduces NO bioavailability and promotes endothelial dysfunction in vascular tissue, whereas in cardiac tissue it contributes to hypertrophy and uremic cardiomyopathy [56]. The IS–AhR axis therefore functions as a central network amplifier that translates uremic toxin accumulation into systemic redox failure and multiorgan injury in CKD.

**Figure 4:**
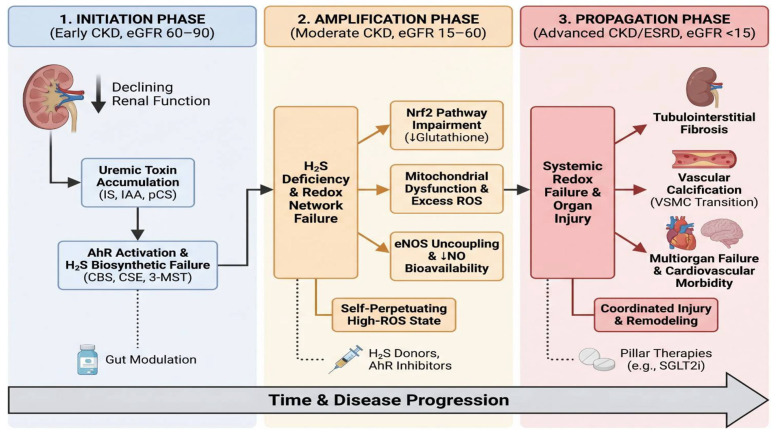
Integrative temporal framework of redox network failure and disease progression in chronic kidney disease. CKD progression is organized into three phases along a temporal axis. In the initiation phase (eGFR 60–90 mL/min/1.73 m^2^), uremic toxin accumulation—including IS, IAA, and pCS—drives AhR-dependent suppression of CBS, CSE, and 3-MST, initiating H_2_S biosynthetic failure. In the amplification phase (eGFR 15–60 mL/min/1.73 m^2^), H_2_S deficiency propagates redox network failure through impaired Nrf2-dependent antioxidant defense, mitochondrial dysfunction with excess ROS generation, and eNOS uncoupling with reduced NO bioavailability, culminating in a self-perpetuating high-ROS state. In the propagation phase (eGFR < 15 mL/min/1.73 m^2^), systemic redox failure drives tubulointerstitial fibrosis, vascular calcification, and multiorgan cardiovascular morbidity. Dashed lines indicate therapeutic intervention points at each phase. Abbreviations: AhR, aryl hydrocarbon receptor; CBS, cystathionine β-synthase; CSE, cystathionine γ-lyase; eGFR, estimated glomerular filtration rate; eNOS, endothelial nitric oxide synthase; H_2_S, hydrogen sulfide; IAA, indole-3-acetic acid; IS, indoxyl sulfate; 3-MST, 3-mercaptopyruvate sulfurtransferase; NO, nitric oxide; Nrf2, nuclear factor erythroid 2-related factor 2; pCS, p-cresyl sulfate; ROS, reactive oxygen species; VSMC, vascular smooth muscle cell.

Figure 4 integrates the mechanistic elements described above into a unified temporal framework of redox network failure and CKD progression, from uremic toxin-driven initiation through redox amplification to systemic organ injury.

## 7. Pathophysiological Consequences of Redox Network Failure

### 7.1. Mitochondrial Dysfunction and Metabolic Reprogramming

Mitochondrial dysfunction is increasingly recognized as a central feature of tubular injury in CKD, particularly under conditions of oxidative stress and impaired sulfur metabolism. Experimental studies have shown that reduced H_2_S availability disrupts mitochondrial homeostasis and weakens antioxidant defense in renal tubular cells. In LLC-PK1 proximal tubular cells, IS markedly reduced endogenous H_2_S production through suppression of CBS, CSE, and 3-MST, accompanied by increased superoxide generation and depletion of intracellular glutathione [9]. These findings suggest that impaired H_2_S biosynthesis contributes to loss of redox buffering capacity in uremic tubular injury. In a separate study using mouse kidneys and human tubular cells, IS induced mitochondrial fragmentation, reduced mitochondrial mass and biogenesis, and impaired both aerobic and anaerobic metabolism, whereas antioxidant treatment partially restored mitochondrial integrity [33]. More recently, Xie et al. demonstrated that AhR activation promoted ubiquitin-dependent degradation of peroxisome proliferator-activated receptor gamma coactivator 1-alpha, leading to suppression of mitochondrial biogenesis, tubular senescence, and renal fibrosis in CKD models [54]. Genetic or pharmacological inhibition of AhR attenuated these mitochondrial abnormalities and improved renal injury phenotypes. Evidence supporting a direct mitochondrial protective role of H_2_S was further provided by Ahmad et al., who showed that the mitochondria-targeted H_2_S donor AP39 preserved ATP generation, reduced mitochondrial ROS production, and attenuated necrotic cell death in oxidatively stressed renal epithelial cells, while partially improving ischemia–reperfusion injury in vivo [110]. In diabetic kidney disease, exogenous H_2_S supplementation also prevented mitochondrial apoptosis by blocking Lon-mediated degradation of mitochondrial superoxide dismutase 2, thereby reducing ROS accumulation and improving renal function [43].

An emerging and mechanistically relevant dimension of tubular redox injury in CKD is ferroptosis, an iron-dependent form of cell death driven by uncontrolled lipid peroxidation. The central regulatory axis governing ferroptosis susceptibility is the system Xc^−^/cysteine–glutathione (GSH)–glutathione peroxidase 4 (GPX4) pathway: cystine import via system Xc^−^ provides cysteine for GSH synthesis, which in turn supports GPX4-mediated reduction of lipid hydroperoxides to non-toxic lipid alcohols [111,112]. In diabetic kidney disease models, tubular solute carrier family 7 member 11 (SLC7A11) and GPX4 expression are reduced alongside decreased GSH and increased lipid peroxidation, and pharmacological ferroptosis inhibition attenuates tubular injury and nephropathy phenotypes [111,113]. In 5/6-nephrectomy CKD rats, tubular GPX4 and SLC7A11 are similarly downregulated with concurrent iron deposition and mitochondrial structural defects, and ferroptosis inhibition reduces fibrosis progression [114]. The connection to H_2_S signaling is mechanistically plausible: H_2_S deficiency in CKD reduces GSH availability and impairs Nrf2-dependent antioxidant gene transcription, potentially lowering the threshold for GPX4 inactivation and ferroptotic cell death. Furthermore, cysteine serves as the shared substrate for both H_2_S biosynthesis via CBS and CSE and for GSH synthesis via the system Xc^−^ pathway. Impaired transsulfuration in CKD may therefore simultaneously compromise H_2_S production and ferroptosis defense, representing a dual vulnerability at the intersection of sulfur metabolism and redox-regulated cell death. Together, these studies support a mechanistic link between impaired H_2_S signaling, mitochondrial dysfunction, and metabolic stress in CKD progression.

### 7.2. Fibrotic Remodeling and Redox-Driven Renal Injury

Progressive fibrosis is a major downstream consequence of sustained oxidative stress and impaired sulfur metabolism in CKD. Experimental studies indicate that H_2_S deficiency promotes fibroblast activation, extracellular matrix accumulation, and inflammatory remodeling through redox-sensitive signaling pathways. In a UUO model, Song et al. demonstrated that plasma H_2_S levels and renal CBS expression were markedly reduced in obstructed kidneys, whereas treatment with NaHS significantly attenuated collagen deposition, extracellular matrix accumulation, and α-smooth muscle actin (α-SMA) expression [23]. In the same study, NaHS also reduced macrophage infiltration and lowered expression of IL-1β, TNF-α, and MCP-1, suggesting that H_2_S modulates both fibrotic and inflammatory responses. Mechanistically, pretreatment with NaHS in NRK-49F renal fibroblasts abolished TGF-β1-induced phosphorylation of SMAD family member 3 (Smad3), p38, c-Jun N-terminal kinase (JNK), and extracellular signal-regulated kinase (ERK), while suppressing expression of collagen I, fibronectin, and α-SMA [23]. Additional UUO studies further showed that exogenous H_2_S reduced oxidative stress and tubulointerstitial fibrosis while preserving antioxidant enzyme activity [115,116]. In chronic obstructive nephropathy, the slow-releasing H_2_S donor GYY4137 reduced epithelial–mesenchymal transition, decreased vimentin and fibronectin expression, and restored E-cadherin expression in obstructed kidneys [117]. Similar findings were observed in HK-2 tubular cells, where NaHS suppressed TGF-β1-induced α-SMA and fibronectin expression through ERK- and β-catenin-dependent pathways while preserving epithelial phenotype markers [20]. More recently, Zhou et al. reported that H_2_S attenuated UUO-induced renal fibrosis through inhibition of NLRP3 signaling and reduction in both M1 and M2 macrophage infiltration [36]. Collectively, these studies support a mechanistic role for H_2_S deficiency in promoting redox-sensitive fibrotic remodeling and tubulointerstitial injury in CKD.

### 7.3. Vascular and Endothelial Dysfunction

Redox triad imbalance alters endothelial signaling through simultaneous disruption of ROS, NO, and sulfur-mediated pathways. Experimental studies indicate that retained uremic solutes directly injure endothelial cells through oxidative mechanisms. In human umbilical vein endothelial cells (HUVECs), IS increased intracellular ROS production by 43% and 74% at concentrations of 125 and 250 μg/mL, respectively, while intracellular glutathione levels declined substantially [47]. Blocking NOX with apocynin reduced IS-induced ROS generation by nearly 71%, whereas inhibition of mitochondrial electron transport or xanthine oxidase produced little effect, indicating that NOX is a major contributor to endothelial oxidative stress in this model [47]. In isolated rat aortic rings, IS (300 μM) reduced acetylcholine-mediated endothelium-dependent relaxation by 42%, accompanied by marked increases in CYP1A1, NOX4, nitrotyrosine, and superoxide expression, together with a 75% reduction in endothelial eNOS staining [66]. Treatment with the AhR antagonist CH223191 restored vascular relaxation and normalized multiple oxidative stress markers, including NOX4 and superoxide levels [66]. These findings place IS–AhR signaling at the center of endothelial redox disturbance and impaired NO bioavailability in CKD.

Persistent ROS excess also reshapes vascular reactivity and endothelial phenotype in experimental vascular disease models. In diabetic db/db mouse aortas, impaired acetylcholine-induced vasorelaxation occurred together with increased vascular ROS accumulation, whereas pharmacological enhancement of eNOS activity restored NO production and improved endothelial responses [118]. Comparable findings were reported in chronic anemia and metabolic stress models, where antioxidant treatment partially recovered endothelium-dependent relaxation in isolated aortic rings [119,120]. Taken together, these studies indicate that sustained oxidative stress and defective NO signaling directly contribute to endothelial dysfunction, altered microvascular regulation, and progressive vascular injury during CKD progression. These endothelial abnormalities may contribute to arterial stiffness, impaired microvascular perfusion, and increased cardiovascular vulnerability observed in CKD populations, although direct causal evidence in humans remains limited.

### 7.4. Vascular Calcification as a Downstream Consequence of Redox Network Failure and Sulfur Metabolic Disruption

Vascular calcification represents a critical but underappreciated downstream consequence of redox network failure in CKD, linking oxidative stress, uremic toxin accumulation, and H_2_S deficiency to active osteogenic reprogramming of vascular smooth muscle cells (VSMCs). H_2_S exerts direct anti-calcific effects through multiple complementary mechanisms: it suppresses phosphate-induced osteoblastic differentiation and mineralization of human aortic VSMCs by inhibiting Pit-1-dependent phosphate uptake and downregulating osteogenic transcription factors including runt-related transcription factor 2 (RUNX2) [121,122]. In calciprotein particle models, H_2_S reduces VSMC calcium loading and oxidative stress through NRF2–NAD(P)H quinone oxidoreductase 1 activation, while in high-glucose and diabetic nephropathy models it prevents elastin degradation and osteogenic transition by suppressing Stat3/cathepsin S signaling through persulfidation of Stat3 Cys259 [123,124]. In calcific aortic valve disease, H_2_S biogenesis and mitochondrial sulfide metabolism are impaired, further supporting the concept that sulfur metabolic disruption contributes directly to pathological mineralization [125].

The accumulation of lanthionine, a CBS/CSE transsulfuration byproduct that rises markedly in CKD, adds a further pro-calcific dimension. At concentrations observed in uremic patients, lanthionine induces bone morphogenetic protein 2 (BMP-2), RUNX2, and alkaline phosphatase expression in endothelial cells, downregulates the pyrophosphate regulator progressive ankylosis protein homolog, and activates ERK1/2 under pro-calcifying conditions, directly promoting vascular mineralization [42]. Clinically, serum lanthionine rises with declining eGFR and parallels CT-based vascular calcium scores, with higher levels associated with elevated pro-inflammatory cytokines in calcified versus non-calcified CKD patients [41]. IS further amplifies this process: IS-treated endothelial cells release microvesicles that induce vascular calcification in vitro and promote Klotho hypermethylation, accelerating the osteogenic transition [126,127]. Together, these findings position vascular calcification as an integrated consequence of the redox triad failure model proposed in this review, in which H_2_S deficiency, lanthionine accumulation, and IS-driven oxidative signaling converge to drive pathological VSMC and endothelial mineralization in CKD.

### 7.5. Integrated Progression: From Redox Failure to Multiorgan Disease

Renal injury, mitochondrial dysfunction, vascular impairment, and inflammation form an interconnected network driven by redox dysregulation. Disruption of coordinated ROS, NO, and sulfur-based signaling propagates across nephron segments and vascular systems, linking oxidative injury, metabolic reprogramming, and endothelial dysfunction into a unified disease process [101].

Feed-forward interactions sustain and amplify this progression. Mitochondrial dysfunction increases ROS production, vascular dysfunction reduces tissue perfusion, and inflammatory signaling maintains fibrotic remodeling [109]. These processes reinforce one another, transforming localized molecular disturbances into systemic organ injury.

## 8. Clinical Implications of Redox Network Failure

### 8.1. H_2_S as an Integrative Functional Marker

Reduced H_2_S bioavailability is a consistent feature of CKD and reflects integrated disturbances in redox regulation and sulfur metabolism rather than an isolated biochemical abnormality [39]. Clinical studies indicate that circulating H_2_S levels are reduced by approximately 40–60% in CKD compared with healthy controls and correlate with declining eGFR, increasing proteinuria, and impaired tubular function [39]. These observations suggest that H_2_S captures dimensions of disease severity not fully represented by conventional clinical markers.

H_2_S plays a central role in regulating vascular tone, tubular transport, and renal hemodynamics, linking redox balance to functional adaptation [128]. Reduced H_2_S bioavailability reflects impaired antioxidant capacity, heightened inflammatory signaling, and diminished adaptive responses, indicating a state of increased biological vulnerability rather than structural damage alone [5]. Experimental studies further demonstrate that restoration of H_2_S signaling attenuates fibrosis and improves renal outcomes in CKD models [34].

Despite these associations, clinical application remains limited. Most available data are observational, and variability in measurement techniques and the presence of multiple circulating sulfur pools limit standardization and reproducibility [129]. Accordingly, H_2_S is best considered a translational indicator of redox network status rather than a routine clinical biomarker at present.

A critical and underappreciated challenge concerns the analytical validity of H_2_S measurements themselves. Plasma sulfide exists across multiple distinct pools—free dissolved H_2_S, acid-labile sulfur bound to proteins and metal centers, and sulfane sulfur species—each with different biological relevance, yet most clinical studies measure only one pool without clearly specifying which [130,131]. H_2_S is highly volatile and rapidly oxidized under aerobic conditions, meaning that losses during sample collection, storage, and processing can introduce systematic underestimation that is difficult to distinguish from true biological deficiency [132]. Methodological discrepancies compound this problem: the widely used methylene blue colorimetric assay substantially overestimates free sulfide by reacting with bound sulfur pools, whereas monobromobimane–HPLC methods, although more sensitive, yield substantially different values depending on pretreatment conditions and alkylating agent choice [133,134]. Electrode-based approaches require strongly alkaline conditions that can liberate acid-labile sulfur, further confounding speciation, while LC-MS/MS with isotope-labeled internal standards currently provides the most accurate quantification but remains technically demanding for routine clinical use [135,136]. Reported plasma H_2_S concentrations across clinical studies consequently span nanomolar to hundreds of micromolar ranges, reflecting methodological heterogeneity as much as biology [130]. These limitations do not invalidate the consistent directional finding of reduced sulfide bioavailability in CKD, but they underscore that cross-study comparisons and absolute threshold values should be interpreted with caution until standardized, pool-specific analytical protocols are adopted.

### 8.2. Cardiorenal and Metabolic Implications

H_2_S deficiency has implications that extend beyond renal function and encompasses cardiovascular risk in CKD [39]. Reduced H_2_S signaling impairs endothelial protection, promotes oxidative stress, and disrupts vascular tone, thereby contributing to endothelial dysfunction, hypertension, and atherosclerosis [39,101]. These effects support a cardiorenal framework in which redox network disruption links renal impairment to systemic vascular injury.

Alterations in sulfur metabolism further contribute to metabolic instability. CKD is characterized by hyperhomocysteinemia and impaired transsulfuration, reflecting disrupted coupling between homocysteine metabolism and H_2_S production [8]. This imbalance promotes oxidative stress, inflammation, and endothelial dysfunction [47,102]. However, some sulfur metabolites appear to correlate more closely with renal function than with independent cardiovascular risk, suggesting that they may serve primarily as markers of disease severity rather than direct causal mediators [137].

These findings support the concept of CKD as a systemic cardiorenal–metabolic disorder driven in part by redox network dysfunction. H_2_S signaling intersects with NO pathways, RAAS activation, oxidative stress, and gut-derived toxins, positioning H_2_S deficiency as an integrative indicator of systemic dysregulation [101].

Two underappreciated sources of biological variability in sulfur-mediated redox regulation are sex and age, both of which have direct implications for precision medicine approaches in CKD. Estrogen upregulates CBS expression in vascular endothelial and smooth muscle cells through estrogen receptor-dependent promoter activation, enhancing H_2_S-mediated vasodilation and contributing to the relative cardiovascular protection observed in premenopausal women [138,139]. Consistent with this, female prediabetic rats demonstrate better-preserved endothelial function associated with higher H_2_S-producing enzyme expression in perivascular tissue, while circulating sulfane sulfur pools show sex-specific reductions in human cardiovascular disease [140,141]. With advancing age, renal H_2_S levels decline despite compensatory upregulation of CBS and CSE in some tissues, and this decline is functionally significant: exogenous H_2_S restores AMP-activated protein kinase (AMPK) activity, reduces senescence-associated secretory phenotype markers, and attenuates age-related renal fibrosis, while dietary restriction-mediated increases in CBS and CSE expression delay renal senescence in aged rats [142,143]. At the cellular level, H_2_S deficiency accelerates senescence through loss of Keap1 S-sulfhydration and Nrf2-dependent antioxidant defense, while restoration of H_2_S signaling improves mitophagy and mitochondrial function in aging glomerular mesangial cells via the AMPK–unc-51-like autophagy activating kinase 1 (ULK1)–PTEN-induced kinase 1–parkin axis [5,144]. These findings suggest that the magnitude of H_2_S deficiency and its pathological consequences in CKD may vary substantially by sex and age, and that therapeutic strategies targeting sulfur metabolism should account for these biological variables in trial design and patient stratification.

### 8.3. Clinical Translation and Future Directions

Despite substantial mechanistic evidence linking H_2_S deficiency to redox dysregulation, its clinical role remains incompletely defined. Measurement represents a major challenge due to the presence of multiple circulating sulfur pools and variability in analytical methods [129]. In addition, most available human data are cross-sectional, with limited prospective studies evaluating longitudinal changes or predictive value for clinical outcomes [39].

Future research should prioritize validation of H_2_S as a prognostic marker in well-characterized CKD cohorts and development of standardized assays capable of distinguishing biologically relevant sulfur species. Integration of H_2_S with redox-related biomarkers and uremic toxin profiles may improve risk stratification and provide mechanistic insight into disease progression.

Importantly, interventional studies are required to determine whether modulation of H_2_S signaling translates into improved renal and cardiovascular outcomes. Such studies will be essential to define the therapeutic relevance of targeting redox network dysfunction and to establish H_2_S-based strategies within precision medicine approaches for CKD. In this context, future studies should also address whether H_2_S deficiency is uniformly pathogenic across CKD stages or whether context-dependent biphasic effects—modulated by oxygen tension, mitochondrial status, and sulfur substrate availability—define a therapeutic window that varies with disease progression. Characterizing these stage- and context-specific responses will be critical for translating mechanistic insights into targeted interventions and avoiding unintended consequences of H_2_S modulation in heterogeneous CKD populations.

Operationalizing the redox network failure concept clinically will require a multimodal biomarker strategy that extends beyond H_2_S alone. A panel approach integrating markers across the three redox axes could capture the coordinated nature of network disruption: oxidative stress markers including malondialdehyde, F2-isoprostanes, and advanced oxidation protein products reflect ROS axis status and correlate with CKD stage and cardiovascular risk [145,146]; asymmetric dimethylarginine (ADMA), a circulating inhibitor of endothelial NO synthase that accumulates in CKD, provides an accessible index of NO axis impairment and independently predicts cardiovascular outcomes [147]; and sulfur metabolomics encompassing H_2_S, thiosulfate, lanthionine, and homocysteine can characterize the breadth of sulfur axis disruption beyond what H_2_S measurement alone reflects. Uremic toxin profiling of IS and pCS anchors the upstream driver dimension, while emerging mitochondrial stress markers such as growth differentiation factor 15 may capture the downstream bioenergetic consequences of redox network failure [148]. Such a composite panel would not only improve risk stratification beyond conventional eGFR and proteinuria but also provide mechanistically interpretable endpoints for interventional studies targeting redox network restoration—a prerequisite for moving beyond the surrogate marker limitations that have constrained prior antioxidant trials in CKD.

### 8.4. Unresolved Controversies and Critical Knowledge Gaps

Despite the mechanistic framework developed in this review, several fundamental uncertainties limit the strength of current conclusions and warrant explicit acknowledgment. First, the relationship between circulating H_2_S and intrarenal sulfur signaling remains incompletely established. Although plasma H_2_S correlates with eGFR and cardiac dysfunction in CKD cohorts [39], renal H_2_S biology is inherently spatiotemporally compartmentalized—CBS, CSE, and 3-MST operate in distinct subcellular and nephron-segment-specific contexts, and tissue-specific dysregulation can occur independently of circulating levels, as demonstrated by the preservation of brain H_2_S-producing capacity despite renal and hepatic enzyme downregulation in CKD [8]. Whether plasma H_2_S accurately reflects the local persulfidation tone, mitochondrial sulfide flux, or glomerular versus tubular H_2_S signaling in individual patients remains unknown, and this uncertainty constrains the interpretability of clinical biomarker data.

Second, it is unclear whether H_2_S donor therapy can selectively restore physiological signaling rather than simply producing broad pharmacological effects. Experimental studies demonstrate that donors such as NaHS inhibit TLR4/NLRP3 signaling and suppress inflammatory cascades through mechanisms that extend beyond H_2_S replacement per se [37], raising the question of whether observed benefits reflect restoration of endogenous sulfur signaling or nonspecific cytoprotective pharmacology. Given the narrow therapeutic window discussed in the following section, and the altered sulfide oxidation capacity in CKD kidneys, the concentration–response relationship between exogenous donor administration and biologically relevant local H_2_S levels in target tissues remains poorly characterized.

Third, AhR inhibition as a therapeutic strategy carries unresolved immunological risks. AhR functions as a homeostatic sensor integrating dietary, microbial, and metabolic ligands to regulate gut barrier integrity, Treg and Th17 differentiation, macrophage polarization, and tonic restraint of microbiota-driven inflammatory signaling in monocytes [149,150]. Systemic AhR blockade therefore risks disrupting immune tolerance, promoting dysbiosis-driven inflammation, and interfering with xenobiotic metabolism—consequences that have not been evaluated in the context of CKD, where immune dysregulation and gut dysbiosis are already present. Selective, tissue- or cell-type-restricted AhR modulation may be necessary to dissociate therapeutic from adverse effects, but the tools to achieve this specificity in vivo remain at an early stage of development [151]. These unresolved questions do not invalidate the proposed framework but define the boundaries of current evidence and the priorities for future mechanistic and translational investigation.

## 9. Therapeutic Implications: Restoring Redox Network Balance in CKD

Recognition of H_2_S deficiency within the framework of redox triad failure supports a shift from nonspecific antioxidant supplementation toward restoration of coordinated redox regulation. In CKD, excessive ROS production and impaired endogenous antioxidant responses contribute to disease progression, while conventional antioxidant strategies have not consistently improved clinical outcomes [100]. H_2_S plays a central role in endogenous redox control through regulation of glutathione availability, antioxidant enzyme activity, and Nrf2 signaling [5]. Therapeutic strategies should therefore aim to restore balance among ROS, NO, and sulfur-based pathways rather than relying on isolated ROS scavenging.

Restoration of H_2_S bioavailability represents a direct downstream approach. In 5/6-nephrectomy models, NaHS improves renal function, reduces oxidative stress, restores antioxidant capacity, and suppresses NF-κB-mediated inflammation and apoptosis [152]. Across experimental kidney disease models, H_2_S donors attenuate oxidative stress, inflammation, autophagy dysregulation, and fibrosis through pathways including TLR4/NLRP3, AMPK–mechanistic target of rapamycin (mTOR), and TGF-β1–Smad3 signaling [153]. Donor compounds differ in pharmacokinetics: NaHS and Na_2_S release H_2_S rapidly, whereas GYY4137 and newer slow-release or activatable donors provide sustained or stimulus-responsive delivery that may better approximate physiological signaling [154,155]. Despite this preclinical promise, the therapeutic application of H_2_S donors is constrained by a narrow concentration-dependent window. In rat models, NaHS at higher doses induces hepatic Complex IV inhibition, increased ROS generation, and apoptosis, whereas lower doses are well tolerated, indicating that the transition from benefit to toxicity occurs within a modest dose range [156]. At the cellular level, sulfide oxidation capacity mediated by SQR determines the threshold below which H_2_S is metabolized safely; loss of SQR activity renders cells susceptible to mitochondrial poisoning at concentrations that would otherwise be tolerated [157]. In CKD, where both H_2_S-producing enzyme activity and sulfur substrate availability are reduced, the metabolic handling of exogenous H_2_S may be substantially altered, potentially narrowing the margin between therapeutic and toxic exposure [158]. Context-dependent risks are further illustrated by the observation that GYY4137 exacerbated cisplatin-induced nephrotoxicity in mice, underscoring that donor safety cannot be assumed across all renal disease contexts [159]. The pharmacological distinctions among donor classes have direct implications for therapeutic design. Fast-releasing inorganic donors such as NaHS generate transient supraphysiological H_2_S peaks that risk cytochrome c oxidase inhibition, whereas slow-hydrolyzing donors such as GYY4137 sustain sub-toxic H_2_S concentrations over hours to days, more closely approximating endogenous production kinetics and demonstrating superior organ protection in ischemia–reperfusion models [160,161]. Glutathione-responsive nanoparticle systems extend this further by providing sustained plasma H_2_S with enhanced cardioprotection compared with both NaHS and GYY4137, illustrating that release kinetics rather than total H_2_S dose may determine therapeutic outcome [161]. Mitochondria-targeted donors incorporating triphenylphosphonium moieties achieve greater than 1000-fold enrichment at the site of ROS generation, conferring potent endothelial and renal epithelial protection at doses orders of magnitude lower than systemic donors, and attenuating cellular senescence through selective modulation of splicing factors [162,163]. Stimulus-responsive donors that activate under pathological conditions such as elevated ROS or reduced pH offer an additional layer of spatial and temporal control, potentially limiting off-target H_2_S release in non-diseased tissue—a property particularly relevant in CKD where systemic sulfide handling is already impaired. Optimal dosing strategies, donor selection, and long-term safety profiling in CKD therefore represent essential prerequisites for clinical translation.

Targeting upstream suppression of endogenous H_2_S production represents a complementary strategy. IS reduces H_2_S synthesis by downregulating CBS, CSE, and 3-MST through AhR-dependent inhibition of Sp1 activity, accompanied by glutathione depletion and increased oxidative stress [9]. While H_2_S donors can partially restore redox balance, approaches that reduce uremic toxin burden or modulate AhR signaling may more effectively address the underlying drivers of H_2_S deficiency.

A broader translational concern is whether H_2_S-based strategies may encounter the barriers that have limited conventional antioxidant therapies in CKD. Clinical trials of nonspecific antioxidants including bardoxolone methyl demonstrated that indiscriminate modulation of redox pathways can produce unexpected off-target effects, and that surrogate biochemical endpoints do not reliably predict hard clinical outcomes [4]. H_2_S donors share some of these vulnerabilities: they act on multiple pathways simultaneously, lack validated clinical biomarkers for target engagement, and have not yet been tested in prospective CKD outcome trials. The narrow therapeutic window described above, combined with altered sulfide oxidation capacity in CKD kidneys, means that dosing strategies validated in experimental models may not translate directly to patients with advanced renal impairment. Avoiding the failures of prior antioxidant approaches will therefore require not only improved donor chemistry but also rigorous pharmacokinetic characterization in CKD populations, identification of responsive patient subgroups, and selection of mechanistically justified clinical endpoints that reflect redox network restoration rather than isolated oxidative stress markers.

An integrated therapeutic framework should therefore combine downstream restoration of H_2_S signaling with upstream modulation of uremic toxin pathways, together with broader strategies aimed at re-establishing redox network balance. Future studies should prioritize in vivo assessment of H_2_S bioavailability, integration with uremic toxin profiling, and evaluation of renal and cardiovascular outcomes in well-designed CKD trials. These efforts will be essential to determine whether targeting redox network dysfunction can be translated into clinically meaningful therapeutic benefit.

Integration of H_2_S-targeted strategies with established CKD therapeutics remains an important but largely unaddressed translational question. Sodium-glucose cotransporter 2 inhibitors improve mitochondrial biogenesis and reduce NOX4-driven ROS through AMPK- and sirtuin 1-dependent mechanisms, while glucagon-like peptide-1 (GLP-1) receptor agonists and non-steroidal mineralocorticoid receptor antagonists confer overlapping mitochondrial and anti-inflammatory renal benefits [164,165]. Since these pathways converge on mitochondrial ROS reduction and Nrf2-related antioxidant defenses—which directly intersect with H_2_S-mediated redox regulation—it is plausible that H_2_S deficiency may limit their full therapeutic potential, or that combination approaches could produce additive renoprotection. However, no direct evidence currently links any of these drug classes to renal CBS, CSE, or 3-MST activity or H_2_S bioavailability in CKD, representing a critical knowledge gap that future mechanistic and clinical studies should address.

## 10. Conclusions

Growing evidence suggests that H_2_S deficiency in CKD reflects a broader impairment of sulfur-mediated redox regulation rather than simple antioxidant depletion. Cellular and animal studies consistently show downregulation of CBS, CSE, and 3-MST, disruption of protein persulfidation and mitochondrial sulfide oxidation, and suppression of H_2_S biosynthesis through IS–AhR–Sp1-dependent mechanisms. These molecular disturbances interact with oxidative stress, mitochondrial dysfunction, and inflammatory signaling across renal, vascular, and cardiac experimental models.

At a systems level, concurrent dysregulation of ROS, NO, and H_2_S signaling appears to amplify endothelial dysfunction, fibrotic remodeling, and inflammatory activation in a self-reinforcing manner, supporting the concept of CKD as a condition of coordinated redox imbalance. Loss of sulfur-mediated signaling may therefore contribute to progression of renal and cardiovascular injury, though many proposed mechanisms still lack direct validation in human CKD tissues. Expanding the scope of this framework to incorporate reactive sulfur species beyond H_2_S, cell-type-specific vulnerabilities, and stage-dependent metabolic heterogeneity will be important for refining its translational relevance. Additional translational and clinical studies will be needed to define the therapeutic potential of H_2_S-targeted approaches in CKD.

## Data Availability

No new data were created or analyzed in this study. Data sharing is not applicable to this article.

## Abbreviations

The following abbreviations are used in this manuscript:

ADMA	asymmetric dimethylarginine
AhR	aryl hydrocarbon receptor
AHRR	aryl hydrocarbon receptor repressor
AKI	acute kidney injury
AMPK	AMP-activated protein kinase
AP-1	activator protein 1
ARNT	aryl hydrocarbon receptor nuclear translocator
ASC	apoptosis-associated speck-like protein containing a CARD
ATM	ataxia telangiectasia mutated kinase
ATP	adenosine triphosphate
BMP-2	bone morphogenetic protein 2
CBS	cystathionine β-synthase
CKD	chronic kidney disease
CO	carbon monoxide
CREB	cAMP response element-binding protein
CSE	cystathionine γ-lyase
CYP1A1	cytochrome P450 family 1 subfamily A member 1
CysSSH	cysteine persulfide
eGFR	estimated glomerular filtration rate
eNOS	endothelial nitric oxide synthase
ERK	extracellular signal-regulated kinase
ESRD	end-stage renal disease
ETHE1	ethylmalonic encephalopathy protein 1
GAPDH	glyceraldehyde-3-phosphate dehydrogenase
GLP-1	glucagon-like peptide-1
GPX4	glutathione peroxidase 4
GSH	glutathione
GSSH	glutathione persulfide
H_2_S	hydrogen sulfide
HDAC	histone deacetylase
IAA	indole-3-acetic acid
IS	indoxyl sulfate
JNK	c-Jun N-terminal kinase
Keap1	Kelch-like ECH-associated protein 1
MAPK	mitogen-activated protein kinase
MCP-1	monocyte chemoattractant protein-1
3-MST	3-mercaptopyruvate sulfurtransferase
mTOR	mechanistic target of rapamycin
NAD^+^	nicotinamide adenine dinucleotide (oxidized)
NADH	nicotinamide adenine dinucleotide (reduced)
NADPH	nicotinamide adenine dinucleotide phosphate
NF-κB	nuclear factor kappa B
NLRP3	NOD-like receptor family pyrin domain containing 3
NO	nitric oxide
NOX	NADPH oxidase
NOX4	NADPH oxidase 4
Nrf2	nuclear factor erythroid 2-related factor 2
pCS	p-cresyl sulfate
RAAS	renin–angiotensin–aldosterone system
ROS	reactive oxygen species
RSS	reactive sulfur species
RUNX2	runt-related transcription factor 2
SLC7A11	solute carrier family 7 member 11
α-SMA	alpha-smooth muscle actin
Sp1	specificity protein 1
SQR	sulfide:quinone oxidoreductase
TGF-β1	transforming growth factor-beta 1
TLR4	Toll-like receptor 4
TNF-α	tumor necrosis factor-alpha
TST	thiosulfate sulfurtransferase
ULK1	unc-51-like autophagy activating kinase 1
UUO	unilateral ureteral obstruction
VSMC	vascular smooth muscle cell
